# Community-Engaged Research and Workforce Education in Mobile Health: Tips for Successful Replication

**DOI:** 10.1093/geroni/igaf122.271

**Published:** 2025-12-31

**Authors:** Lana Sargent, Barbara Resnick

## Abstract

Mobile clinics have demonstrated success in addressing community needs due to their flexibility and responsiveness. Utilizing a mobile health platform to provide workforce education and integrate community-based research can lead to sustained relationship building. Community-based workforce education and research requires engaging diverse partners, capacity building for student learning outcomes, and trust building to facilitate research participation. To advance the science of community-engaged research (CEnR), we convene leading scholars who will provide insight into the inherent issues of engaging the community in research and education with strategies for overcoming obstacles. The first presentation will present how the Mobile Health and Wellness (MHWP) program was conceived and factors leading to its sustainability over 12 years of continuous growth in scope of service, education, and research along with resources for replication. The second will demonstrate how to gain the Institute for Healthcare Improvement as an Age-Friendly Health System--Committed to Care Excellence by providing reliable practice assessing and acting on the 4Ms of age-friendly care in a community-based health and wellness program. The third will present results from a mixed methods study evaluating the training programs’ results hosted at MHWP based on age-friendly care with medically underserved communities. The final presentation will present outcomes from a transdisciplinary CEnR program to address food insecurity in the mobile clinic setting, uncovering the effects of diet and social risk factors for cardiovascular disease. This symposium concludes with reflections about meaningful and authentic academic-community partnerships that align with supporting and building bridges between aspiring and existing community-engaged scholars.

